# Initial Experience With the Next-Generation Resolute Onyx Zotarolimus-Eluting Stent in Symptomatic Intracranial Atherosclerotic Disease

**DOI:** 10.3389/fneur.2020.570100

**Published:** 2020-09-30

**Authors:** Ameer E. Hassan, Mahmoud H. Mohammaden, Rani Ramsey Rabah, Wondwossen G. Tekle

**Affiliations:** ^1^Clinical Research Department, Valley Baptist Medical Center – Harlingen, Harlingen, TX, United States; ^2^Department of Neurology, School of Medicine, University of Texas Rio Grande Valley, Edinburg, TX, United States; ^3^Department of Neurology and Psychiatry, College of Medicine, South Valley University, Qena, Egypt; ^4^Department of Neurology, Marcus Stroke and Neuroscience Center, Grady Memorial Hospital, University School of Medicine, Atlanta, GA, United States

**Keywords:** intracranial atherosclerosis, angioplasty, stenting, drug eluting stent, stroke

## Abstract

**Background and Purpose:** Intracranial atherosclerotic disease (ICAD) is a common cause of stroke worldwide. Although there are different endovascular options for the treatment of symptomatic ICAD (sICAD), it is still controversial. Herein, we aim to study the safety and efficacy of a new generation of drug-eluting balloon-mounted stent (DES); Resolute (R) onyx DES in the treatment of sICAD.

**Methods:** A prospectively maintained neuroendovascular procedures database in a high-volume comprehensive stroke center was reviewed from October 2019 through January 2020. Patients were included if they had sICAD (≥70% stenosis), failed medical management, and underwent intracranial stenting with R-onyx DES. Technical success was defined as the ability to deploy the device at the desired location and achievement of <30% residual stenosis. The primary outcome was the occurrence of complications within 72 h of the procedure (strokes, ischemic or hemorrhagic; and mortality). Secondary outcomes included rates of symptomatic and angiographic recurrence within 6 months of the procedure.

**Results:** A total of 18 consecutive patients (mean age, 66.6 years; 44.4% were females and 94.4% were Hispanic) were eligible for the analysis. Indication for treatment was recurrent strokes in 13 and recurrent transient ischemic attack (TIA) in 5. A total of 22 symptomatic lesions with a mean baseline stenosis percent (84.9 ± 9.6) were treated using 23 R-onyx DES in 19 procedures. All procedures were done under general anesthesia with 100% technical success, and no reported periprocedural strokes or death. Among 13 patients who had clinical follow-up, 1 (7.7%) patient had TIA. There were no reported ischemic or hemorrhagic strokes. Angiographic follow-up for 9 (50%) patients showed no in-stent restenosis.

**Conclusion:** The use of R-onyx DES in the treatment of sICAD is safe with high technical success rates. Large prospective multicenter trials with long-term follow-up are warranted.

## Introduction

Intracranial atherosclerotic disease (ICAD) is a common cause of stroke worldwide, with variable prevalence among different races (1). Endovascular treatment (ET) has been controversial since the results of randomized clinical trials (RCTs) that compared medical treatment (MT) vs. ET, Stenting vs. Aggressive Medical Management for Preventing Recurrent stroke in Intracranial Stenosis (SAMMPRIS) (2), and Vitesse Intracranial Stent Study for Ischemic Stroke Therapy (VISSIT) (3) trials were terminated in advance because ET groups showed a significant increase in perioperative complications. On the other hand, a single-center RCT in China (4) found that ET could be a safe and efficient treatment modality for carefully selected patients with ICAD due to middle cerebral artery stenosis. The Wingspan Stent System Post Market Surveillance (WEAVE) trial (5) reported improved safety of intracranial stenting with a periprocedural event rate of stroke or death of 2.6% when stenting was performed using the Food and Drug Administration (FDA)–approved indication and by experienced operators. Different types of stents can be used in intracranial stenting: self-expandable stent (SES) (5, 6) and drug-eluting balloon-mounted stents (DES) (7–11). The former has a lower radial force; therefore, it is less suitable to achieve the ideal luminal dilatation, especially in those with calcified lesions and has higher rates of in-stent restenosis (ISR) (12, 13). Although DES reduces the risk of ISR (14) “by delivering an antiproliferative drug that prevents neointimal hyperplasia,” the delivery system is usually stiff, hence navigation along tortuous intracranial vasculature could be difficult. Two generations of DES have evolved according to their antiproliferative agents, the first generation (paclitaxel/sirolimus-eluting stents) and the second generation (everolimus/zotarolimus-eluting stents), where the stent is more flexible than the latter.

In the present study, we aim to evaluate the safety and efficacy of a new generation of DES, Resolute (R) onyx DES (Medtronic, Santa Rosa, CA) in the treatment of sICAD.

## Materials and Methods

### Patient Selection

We retrospectively reviewed a prospectively maintained neuroendovascular procedures database in a comprehensive stroke center from October 2019 through January 2020. Patients were included in the analysis if they had sICAD: ≥70% intracranial stenosis, recurrent strokes, or transient ischemic attacks (TIAs) in the territory of the affected artery despite aggressive MT^2^ and baseline modified Rankin Scale (mRS) ≤3. The Institutional Review Board approved the study and written informed consent was obtained from all participants to use the off-label stent.

### Device Description

R-onyx DES (Medtronic) Zotarolimus-Eluting Coronary Stent System consists of a balloon-expandable, intracoronary DES premounted on a Rapid Exchange or an Over-the-Wire stent delivery system. R-onyx DES is manufactured from a composite material of cobalt alloy and 90% platinum−10% iridium alloy and is formed from a single wire bent into a continuous sinusoid pattern that then laser fused back onto itself. The stents are available in multiple lengths and diameters. The delivery system has two radiopaque markers to aid in the placement of the stent during fluoroscopy and is compatible with 0.014-in. (0.36-mm) guidewires and 1.42-mm (5 Fr/0.056 in.) minimum inner diameter guide catheters. The strut thickness is 81 μm. R-Onyx has a swaged shape and a larger strut width-to-thickness ratio than the old generation (Resolute). Zotarolimus dose density and polymer are identical to the Resolute DES; however, because of the modified stent geometry, the overall drug load is slightly reduced in most sizes of the R-Onyx DES (15).

### Endovascular Procedure

The decision to pursue with endovascular treatment was based on multidisciplinary discussion between vascular neurologists and neurointerventionalists. All procedures were performed under general anesthesia. A dose of 325 mg of acetylsalicylic acid (ASA) and 75 mg of clopidogrel was given at least 3 days before ET. Platelet function was assessed by P2Y12 reaction units (PRU) test with a target of 60–200; if it was above 200, a loading dose (180 mg) of ticagrelor was given then the patient was started on ticagrelor 90 mg BID and ASA 81 mg daily and discontinued clopidogrel. Femoral access was used for anterior circulation lesions, whereas posterior circulation lesions were approached through radial access. Two types of guiding catheters were used: ballast long sheath (Balt, Irvine, CA, USA) and Neuron 088 Max (Penumbra, Alameda, CA, USA), and the choice between the two devices was depending on operator's preference. Similarly, two types of distal access catheters were used: Navien 5Fr (Medtronic, Irvine, CA, USA) and Sofia 5Fr (MicroVention, Tustin, CA, USA). During the intervention, all patients were heparinized to activated clotting time from 250 to 300 s. A radiologic examination of the targeted vessel was performed using a biplane angiographic system (Innova IGS 630; GE Healthcare, Chalfont St Giles, UK), the vessel diameter adjacent to the stenosis and the diameter and length of the stenosis were determined for proper selection of the stent size. The degree of percent stenosis was determined as follows: percent stenosis = [(1 – (D_stenosis_/D_normal_))] × 100, where D_stenosis_ = the diameter of the artery at the site of the most severe stenosis and D_normal_ = the diameter of the proximal normal artery (16).

Under a road map, the vessel distal to the stenosis was catheterized with a microwire; in case of near occlusion of the targeted vessel, a pre-dilatation with a balloon (Gateway; Stryker, Kalamazoo, MI, USA) was performed, then the R-onyx DES was deployed at nominal pressure (12 atm) with a manometer. After deflation and withdrawal of the balloon catheter, a final DSA run was carried out to confirm stent deployment at the targeted stenosis and to exclude complications.

Dual antiplatelet therapy with either ticagrelor 90 mg plus ASA 81 mg or clopidogrel 75 mg plus ASA 81 mg continues indefinitely after the procedure.

### Outcome and Follow-Up

The primary outcome was the incidence of strokes (ischemic, hemorrhagic) and death within 72 h post-stenting which was assessed clinically before patient discharge by a vascular neurologist and radiologically through head CT. Technical success was defined as the ability to deploy the device at the desired location and achievement of <30% residual stenosis. Patients underwent clinical and angiographic follow-up within 6 months after the procedure to assess for symptomatic and angiographic recurrence.

### Statistical Analysis

Categorical variables were expressed as frequencies and percentages. After normality testing through Shapiro–Wilk, continuous variables were expressed as mean ± SD for parametric and as median for non-parametric variables. The analysis was performed using SPSS 26 software (IBM, Armonk, NY, USA).

## Results

**Figure 1:**
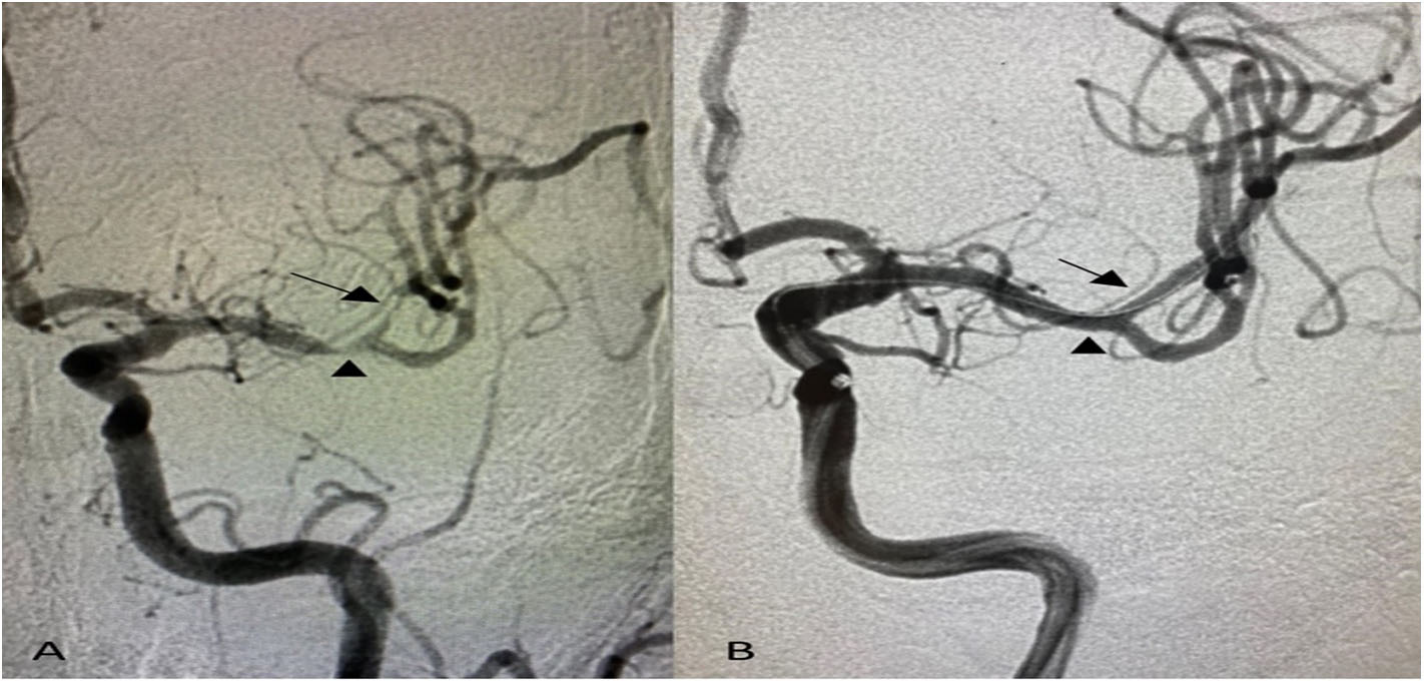
Anterior–posterior projection of digital subtraction angiography shows **(A)** tandem intracranial significant stenoses of the left middle cerebral artery; distal M1 segment (arrowhead) and superior division of M2 segment (arrow), **(B)** post-angioplasty and stenting with no residual stenosis using 2 Resolute Onyx Drug-Eluting Stents 2 × 8 mm.

A total of 18 patients were eligible for the analysis. The mean age was 66.6 ± 12 years, 44.4% were females, and 94.4% were Hispanic. Stroke risk factors included hypertension in all patients (100%), diabetes mellitus in 12 (66.7%), hyperlipidemia in 9 (50%), and current cigarette smoking in 2 (11.1%). Moreover, 72.8% had recurrent strokes in the territory of the affected blood vessel, and 27.8% had recurrent TIA. Nineteen symptomatic lesions were treated with a mean baseline stenosis of 84.9 ± 9.6%. Also, 72.7% of the lesions located in the anterior circulation and 27.3% in the posterior circulation. Tandem intracranial lesions occurred in 16.7% of patients (Figure 1, Table 1).

**Table 1:** Patients' demographics, risk factors, and lesion characteristics.

**All patients *n* (%)**	***n* = 18**
Age (years) mean ± SD	66.6 ± 12
Female	8 (44.4)
**Race**	
Hispanic	17 (94.4)
White	1 (5.6)
Hypertension	18 (100)
Diabetes mellitus	12 (66.7)
Hyperlipidemia	9 (50)
Current cigarette smoking	2 (11.1)
Recurrent stroke	13 (72.2)
Recurrent TIA	5 (27.8)
**Total lesions** ***n*** **(%)**	***n*** **=** **22**
**Stenosis location**	
Anterior circulation:	16 (72.7)
Supraclinoid ICA	3 (13.6)
Cavernous ICA	3 (13.6)
Petrous ICA	2 (9.1)
**Middle cerebral artery**	
M1 segment	4 (18.2)
M2 segment	4 (18.2)
Posterior circulation:	6 (27.3)
Vertebral artery V4 segment	3 (13.6)
Basilar artery	2 (9.1)
Posterior cerebral artery	1 (4.5)
Tandem intracranial lesions per subject	3 (16.7)
Baseline stenosis percent (%) mean ± SD	84.9 ± 9.6

*ICA, internal carotid artery; TIA, transient ischemic attack*.

A total of 19 procedures were performed under general anesthesia with median fluoroscopy time 12.2 (10–18.3) min; mean contrast volume, 50.4 ± 24 ml. The median time of stenting from the last stroke was 4.5 (1.8–67.5) days, with 10 (52.6%) patients being treated within 7 days of the last stroke. The lesion was accessed through a femoral puncture in 15 (78.9%) and radial puncture in 4 (21.1%) of the procedures. Pre-dilatation with a balloon was performed in four (21.1%) procedures. In-stent thrombosis occurred in one procedure, which was resolved with intra-arterial tirofiban without complications. A total of 23 stents were deployed, 1.3 per procedure and 9 (39.1%) were 2 × 8 mm in size. The overall procedural success rate was 100%, with no reported periprocedural ischemic or hemorrhagic strokes and death (Table 2).

**Table 2:** Procedural characteristics and outcome.

**Procedural characteristics *n* (%)**	***n* = 19**
Time of stenting from the last stroke (days) median (IQR)	4.5 (1.8–67.5)
Procedures ≤ 7 days of the last stroke	10 (52.6)
**Arterial access**	
Femoral	15 (78.9)
Radial	4 (21.1)
General anesthesia	19 (100)
IA-tirofiban	1 (5.3)
R-onyx DES:	*n* = 23
Stent per procedure	1.3
**Stent size:**	
2 × 8 mm	9 (39.1)
**Number of lesions planned for treatment**	
1	16 (84.2)
2	3 (15.8)
Pre-stenting balloon angioplasty	4 (21.1)
Technical success	19 (100)
No residual stenosis	15 (78.9)
Non-significant residual stenosis	4 (21.1)
Total contrast (ml) mean ± SD	50.4 ± 24
Fluoroscopy time (min) median (IQR)	12.2 (10–18.3)
**Outcome**	
**Procedural complication**	
In-stent thrombosis	1 (5.3)
Ischemic stroke	0 (0)
Intracerebral hemorrhage	0 (0)
**Follow-up**	
**Clinical (13 patients)**	
TIA	1 (7.7)
Ischemic and hemorrhagic strokes	0 (0.0)
**6-month angiogram (9 patients)**	
In-stent stenosis	0 (0.0)

*IQR, interquartile range; TIA, transient ischemic attack*.

### Clinical and Angiographic Follow-Up

Among 13 patients who had clinical follow-up, one (7.7%) patient had transient ischemic attack in the same territory of the treated artery 2 months after the procedure. There were no reported ischemic/hemorrhagic strokes or medication-related complications. Nine (50%) patients underwent digital subtraction angiography on follow-up and showed no ISR (Table 2).

## Discussion

We successfully treated 18 patients with sICAD using the Medtronic Resolute Onyx drug-eluting balloon-mounted stent. There was no periprocedural stroke or death within 72 h of stenting. Moreover, there were no reported cases of ISR among patients who had 6-month angiographic follow-up.

Currently, the Wingspan stent (Stryker) is the only FDA-approved stent for the treatment of sICAD under the strict indications applied in the WEAVE trial (5). Other applications of ET for sICAD include balloon angioplasty alone (17) or followed by SES (6), DES (7–11), and more recent use of a drug-eluting balloon (18). Angioplasty alone without stent placement is associated with higher rates of restenosis and procedural complications due to the elastic recoil of blood vessels and the risk of dissection (19).

The present case series reported high safety and technical success in the treatment of sICAD using the most recent generation of DES. R-onyx DES not only delivers zotarolimus with “a more potent with less systemic side effect than first-generation antiproliferative” (20) but also the stent geometry is different from the old generation [Resolute and previously studied Resolute Integrity (21)]. It has a swaged shape and thinner struts that substantially improve stent navigability while maintaining radial strength and lower overall drug load. These advantages, in addition to the use of a more biocompatible polymer, may improve the safety concern about late stent thrombosis (ST) with first-generation DES, which results from incomplete re-endothelialization and persistent fibrin deposition (22).

Periprocedural complications related to DES implantation could result from high-degree stenosis of the target vessel, difficult navigability of the stent due to its stiff nature especially in the elderly with tortuous anatomy, and in more distal lesions, deployment of the stent near a perforator and early treatment within 7 days after stroke that may cause reperfusion hemorrhage from the weakened capillary bed and recurrent stroke due to rupture of unstable plaque (23).

**Figure 2:**
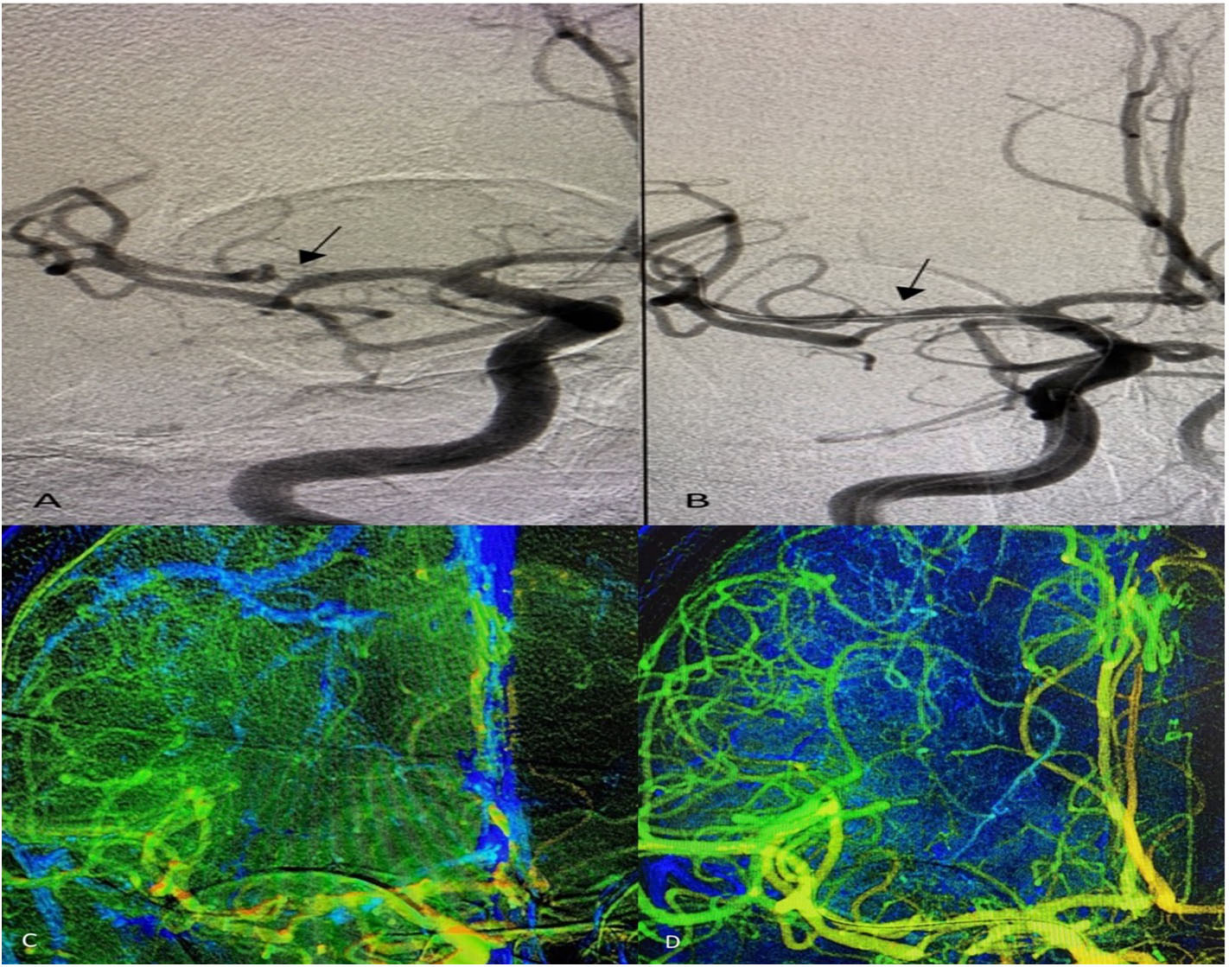
Anterior–posterior projection of digital subtraction angiography shows **(A)** significant stenosis of the right middle cerebral artery–M2 segment (arrow), **(B)** post-angioplasty and stenting with no residual stenosis using Resolute Onyx Drug-Eluting Stents 2 × 8 mm. Two-dimensional parametric parenchymal blood flow demonstrates the difference in blood flow pre- **(C)** and post-angioplasty and stenting **(D)**.

Ye et al. (24) reported a 1.4% incidence of stroke or mortality within 30 days after DES implantation in a group with moderate stenosis <70% that increases to 12.1% if the stenosis was >70%. The present study demonstrated no periprocedural strokes or death with 84.9 ± 9.6% mean baseline stenosis and deployment of the R-onyx DES in different types of lesions; anterior circulation 72.7%, posterior circulation 27.3%, and tandem intracranial lesions 16.7% of procedures (Figure 1). Moreover, more distal lesions (middle cerebral artery M2 segment) within small vessel diameter were treated using the smallest profile of R-onyx DES in the market (2 × 8 mm) with high technical success (Figure 2), and 52.6% of the procedures were performed within 7 days of the last stroke. Also, several studies in the literature (7–9) reported higher rates of periprocedural complications than the present study with a mean age of studied population lower than that demonstrated in our case series.

A major concern for using bare-metal stents has been the rate of ISR which is reported to be ~24 to 45% (13, 25). Previous studies demonstrated several clinical and anatomical predictors of ISR after intracranial stenting including younger age, diabetes mellitus, long lesions, and stenting of small vessels (13, 25, 26). Because ISR is associated with a high rate of recurrent neurologic events (27), DES treatment of sICAD could be more effective especially if associated with the aforementioned risk factors. DES has less incidence of ISR (24); the underlying pathophysiological mechanism of this is inhibition of vessel overreaction after injury and reduction of neointimal thickness which is achieved by delivery of antiproliferative drugs (28).

The technological advances of R-onyx DES provide the unmet needs of intracranial stenting, and the thin strut thickness remains a crucial characteristic in stent platform, demonstrating better device conformability, easier navigability, and lesser strut malapposition. Also, thin struts are associated with low levels of inflammation at the lesion site resulting in rapid and almost complete arterial re-endothelialization and reduced neointimal growth (29). Furthermore, the slim 2-mm stent enables stenting of a small target vessel diameter which is associated with a high incidence of vessel injury, difficult accessibility, and higher rates of ISR. Despite the poor visibility of the slim-profile DES that has ultrathin struts under X-ray images, R-onyx DES was designed with a platinum–iridium core and a cobalt alloy shell to enhance its radiopacity (30).

The new technology of R-onyx DES could be a step forward in the treatment of sICAD if large prospective multicenter trials corroborate our results. The ongoing randomized clinical trial (31) of using dual antiplatelet therapy only for 1 month followed by single antiplatelet therapy after R-onyx DES implantation in patients undergoing percutaneous coronary intervention with high bleeding risk will likely be helpful in determining the adequate duration of antiplatelet regimen in a similar subgroup of sICAD patients.

Our study has all the typical limitations inherent of any single-center retrospective analysis. In addition, the small sample size where only 18 patients were included limits the generalizability of our findings. Similarly, only 50% of patients had follow-up imaging which limited the power to prove the efficacy in preventing ISR. However, the main aim of this study is to identify the periprocedural safety and technical success of the deployment of a new DES that might help in the treatment of sICAD.

## Conclusion

The present case series demonstrate that R-onyx DES can be used in the treatment of sICAD with different types of lesions with high procedural safety and technical success rates. Large multicenter studies are needed to further evaluate procedural safety, restenosis rates, and long-term efficacy.

## Data Availability Statement

The raw data supporting the conclusions of this article will be made available by the authors, without undue reservation.

## Ethics Statement

The studies involving human participants were reviewed and approved by Valley Baptist Medical Center. The ethics committee waived the requirement of written informed consent for participation.

## Author Contributions

AH: study conception, design of the work, and critical revision of the article. MM: study conception, design of the work, interpretation of data, and drafting of the article. RR: data acquisition and critical revision of the article. WT: critical revision of the article. All authors gave final approval of the version to be published.

## Conflict of Interest

AH: Consultant/Honorarium/Scientific Advisor: Medtronic, Stryker, Penumbra, Genentech, Viz, Balt, Microvention, GE Healthcare, Scientia, and Cerenovus. The remaining authors declare that the research was conducted in the absence of any commercial or financial relationships that could be construed as a potential conflict of interest.
